# Temporal variation in selection on male and female traits in wild tree crickets

**DOI:** 10.1002/ece3.2105

**Published:** 2016-06-16

**Authors:** Kyla Ercit

**Affiliations:** ^1^ Department of Ecology and Evolutionary BiologyUniversity of Toronto at Mississauga3359 Mississauga Rd NMississaugaOntarioCanadaL5L 1C6

**Keywords:** *Isodontia mexicana*, *Oecanthus nigricornis*, predation, sexual dimorphism, temporal variation in selection, viability selection

## Abstract

Understanding temporal variation in selection in natural populations is necessary to accurately estimate rates of divergence and macroevolutionary processes. Temporal variation in the strength and direction of selection on sex‐specific traits can also explain stasis in male and female phenotype and sexual dimorphism. I investigated changes in strength and form of viability selection (via predation by wasps) in a natural population of male and female tree crickets over 4 years. I found that although the source of viability stayed the same, viability selection affected males and females differently, and the strength, direction and form of selection varied considerably from year to year. In general, males experienced significant linear selection and significant selection differentials more frequently than females, and different male traits experienced significant linear selection each year. This yearly variation resulted in overall weak but significant convex selection on a composite male trait that mostly represented leg size and wing width. Significant selection on female phenotype was uncommon, but when it was detected, it was invariably nonlinear. Significant concave selection on traits representing female body size was observed in some years, as the largest and smallest females were preyed on less (the largest may have been too heavy for flying wasps to carry). Viability selection was significantly different between males and females in 2 of 4 years. Although viability selection via predation has the potential to drive phenotypic change and sexual dimorphism, temporal variation in selection may maintain stasis.

## Introduction

Understanding the pace of phenotypic selection in natural populations is important to accurately calculate the rates of divergence and macroevolutionary processes, and to estimate how quickly populations can respond to change, man‐made or otherwise (Siepielski et al. 2009). Selection analysis, the measurement of the relationship between phenotypic traits and relative fitness in a population (Lande and Arnold 1983), is an important tool for standardizing estimates of natural selection in order to detect such patterns. Since Lande and Arnold's (1983) landmark paper on quantifying selection using selection analyses, thousands of studies have used these methods, and several meta‐analyses have compiled and compared selection gradients (Hoekstra et al. 2001; Kingsolver et al. 2001; Hereford et al. 2004; Kingsolver and Diamond 2011). However, compiled estimates of selection predict faster microevolution than is generally observed (Kinnison and Hendry 2001). This mismatch could be due to over‐estimating selection in the long term (over several generations) when using selection gradients measured in short term (over a single generation or single breeding season), as estimates of selection can be greatly affected by the duration of the episode of selection (Hoekstra et al. 2001). Longer‐term studies of selection on natural populations are valuable as they provide information on how the strength and direction of selection is affected by timescale (Kingsolver et al. 2001; Kingsolver and Pfennig 2007). In longer intervals of time, such as over several generations, temporal variation in the strength and direction of selection and variation in which traits are under selection can reduce the magnitude of net selection across years, which would result in slower evolution than predicted from a single episode of selection (Siepielski et al. 2009, 2011; but also see Morrissey and Hadfield 2012).

Rates of evolution may be more difficult to predict when dealing with sexually dimorphic traits. Sexually dimorphic traits tend to evolve more slowly than monomorphic traits because males and females of the same species are highly genetically correlated (Lande 1980). Consistently different selection on males and females is necessary to produce sexual dimorphism; thus, temporal variation in the difference in the form and direction of selection between males and females can also slow the evolution of sexual dimorphism (Schulte‐Hostedde et al. 2002; Reimchen and Nosil 2004). If such temporal variation occurs, then traits that produce optimal fitness at different sizes in males and females may remain at an intermediate size that is optimal for neither sex (i.e., intralocus sexual conflict, Cox and Calsbeek 2009).

However, some long‐term studies can reveal consistent selection that can lead to rapid phenotypic change. For instance, rapid evolution has been observed in response to phenotypic selection in populations of stickleback (Bell et al. 2004; Aguirre and Bell 2012), *Anolis* lizards (Losos et al. 2006) and guppies (Reznick et al. 1997). And consistently different selection on male and female house finches (*Carpodacus mexicanus*) can lead to the rapid evolution of sexual dimorphism (Badyaev and Martin 2000; Badyaev 2005). These examples are exceptional although, and usually follow a major change, such as introduction of the species to a new environment or the introduction of a new predator to the current environment. Thus, successive generations would have been affected by new and likely strong selection on the same traits. For populations in more stable environments, this may not be the case.

Populations may experience temporal variation in viability selection as a result of several factors. Highly variable environmental factors can affect the relationship between traits and survival (Kalisz 1986; Grant and Grant 2002; Tarwater and Beissinger 2013). Different phenotypes may also confer survival advantages in one generation but not the next due to changes in major sources of mortality. For example, rotund shell shape in *Physa* snails is advantageous when fish are their dominant predator, but elongate shells are better protection against crayfish predators (DeWitt et al. 2000). Changes in relative predator abundance can also affect the evolution of sexually dimorphic traits, such as spine number in sticklebacks (Reimchen and Nosil 2004). However, it is not well known how selection from the same single source of viability may vary from generation to generation within the same population, or how this variation may affect the evolution of sexually dimorphic traits.

To investigate temporal variation in selection on phenotype and its potential to affect sexual dimorphism, I examined viability selection from a single source of mortality in a natural population of tree crickets, *Oecanthus nigricornis* Walker. I observed selection over 4 years by comparing the phenotypic distribution of surviving crickets to that of prey of a common cricket specialist wasp, *Isodontia mexicana* Saussure. Thus, I compared strength and shape of selection among years and between the sexes. Sex differences in viability selection are particularly of interest in this system because the predator preferentially hunts females (O'Neill and O'Neill 2003, 2009; Ercit 2014), and this differential predation may affect male and female phenotypes differently, potentially producing sexual dimorphism.

## Materials and Methods

### Study organisms

*Oecanthus* tree crickets (Gryllidae) are common in meadows of east and central North America (Capinera et al. 2005). In the area where this study took place, *O. nigricornis* are univoltine, and adults emerge in late July and persist approximately until frost arrives in October. They are typically found in open meadows with *Solidago* spp., *Rubus* spp., and *Daucus* spp., as males use these plants to call from, and females use stems as oviposition sites (Fulton 1915). Adult female tree crickets are larger than males, and males have greatly enlarged forewings (tegmina) which, in females, are less differentiated from the hind wings than in males. Male tegmina are used to produce a calling song that attracts receptive conspecific females (Walker 1957; Bell 1980; Toms 1993; Brown et al. 1996). In pair formation, male *Oecanthus* are mostly stationary, and females are mobile (Fulton 1915; Brown 1999).

*Isodontia mexicana* are common solitary wasps, found throughout southern Canada and the United States, and can be an important predator of *O. nigricornis* (Bohart and Menke 1976; Iwata 1976; O'Neill and O'Neill 2003, 2009; Ercit 2014). Female *I. mexicana* sting and paralyze their prey and carry them back to their nest to provision for their offspring (Iwata 1976). *I. mexicana* are often inhabitants of artificial trap nests (Krombein 1967), so their provisioning behavior is easy to observe. *I. mexicana* take significantly more adult female tree crickets than male (O'Neill and O'Neill 2003, 2009; Ercit 2014), and this is due partly to female‐biased sexual size dimorphism in prey (Ercit 2014), as well, adult female crickets with ovaries heavy with eggs may be easier to catch (Ercit et al. 2014).

### Study site and equipment, and sampling

I monitored *I. mexicana* provisioning behavior from 2009 to 2012 at the University of Toronto Koffler Scientific Reserve (KSR) in King City, ON. Data reported here were collected concurrent to those collected in Ercit (2014) and Ercit and Gwynne (2015), and thus collection methods of these studies are very similar. I sampled wasp prey that was provisioned in artificial trap nests, which were made based on the construction described in (Hallett 2001a,b). Nest blocks were grouped in five stacks of seven, within boxes covered with wooden lids and roofing shingles, and placed on wooden platforms 1 m off the ground.

I collected *O. nigricornis* prey of *I. mexicana* from trap nests and compared these to the overall distribution of crickets collected from the surrounding meadow. Samples were taken approximately weekly, starting every year when the first adult tree cricket appeared in a nest until all wasp provisioning activity had stopped. Prey samples were taken from the most recently provisioned cell of *I. mexicana* nest tunnels, and the entire contents of each recently provisioned cell were taken. Samples of the hunted cricket population were taken for comparison via sweep net on the same day or the previous day as prey samples, from within a 300‐m radius of the wasp nest. These crickets will be hereafter referred to as “survivors.” All samples were housed in small plastic containers and then fixed in 95% ethanol.

### Traits and measurements

Photographs were taken of all cricket samples using an AmScope (Irvine, CA, USA) 5MP microscope digital camera mounted on a Wild Heerbrugg M5A dissecting microscope. I then measured phenotypic traits in the digital photographs using ImageJ (National Institutes of Health, Bethesda, MD, USA) software.

I measured femur length, femur width, tibia length, pronotum length, tegmen width, and head width for all sampled crickets (Table 1). This suite of traits includes both sexually dimorphic (tegmen width and pronotum length) and traits that are monomorphic when accounting for allometry (leg measurements and head width). I included trait tegmen width because tegmina (forewings) are large sound‐producing structures in males, and larger wings may attract more predator attention. I included pronotum length (as a proxy of body size, which is significantly larger in females) because previous results suggest that larger crickets are at higher risk of predation by wasps (Ercit 2014). I included leg measurements because leg size may be related to mobility rate (Kelly et al. 2008). Finally, I included head width because it is a sexually selected trait in male tree crickets (Ercit and Gwynne 2015). To reduce multicollinearity and to increase statistical power, I reduced leg measurements into a single principal component axis that explained 91% of variance. All three leg measurements loaded positively on this axis, and it was mostly influenced by tibia length (50%) and femur length (44%). After this reduction, variance inflation scores were all below 6. Body mass was not measured because all prey crickets necessarily weighed less than survivors as a consequence of paralysis and storage in wasp nests: Paralyzed crickets continued to metabolize their energy stores but could not eat. Instead, I used pronotum length as a proxy as it is the strongest measured predictor of body mass (Ercit 2014).

**Table 1:** Mean (mm) and SEM of male and female traits measured for selection analysis over 4 years

Sex	Trait	Abbreviation	2009	2010	2011	2012	4 years
Males	Pronotum length	PL	2.20 ± 0.019	2.37 ± 0.024	2.36 ± 0.032	2.34 ± 0.020	2.30 ± 0.013
	Head width	HW	1.54 ± 0.008	1.58 ± 0.014	1.61 ± 0.023	1.54 ± 0.009	1.56 ± 0.006
	Tegmen width	TW	6.69 ± 0.056	7.00 ± 0.064	6.92 ± 0.075	6.77 ± 0.064	6.80 ± 0.035
	Tibia length	Components of PC axis “leg size” abbreviated LS	8.54 ± 0.088	9.08 ± 0.106	8.96 ± 0.162	8.74 ± 0.086	8.76 ± 0.053
	Femur length		7.76 ± 0.100	8.29 ± 0.088	8.29 ± 0.108	7.89 ± 0.071	7.96 ± 0.051
	Femur width		1.37 ± 0.010	1.40 ± 0.022	1.41 ± 0.020	1.36 ± 0.012	1.38 ± 0.007
Females	Pronotum length	PL	2.33 ± 0.012	2.51 ± 0.021	2.44 ± 0.023	2.52 ± 0.012	2.43 ± 0.010
	Head width	HW	1.68 ± 0.007	1.74 ± 0.013	1.71 ± 0.019	1.70 ± 0.008	1.70 ± 0.005
	Tegmen width	TW	4.15 ± 0.028	4.38 ± 0.031	4.33 ± 0.058	4.32 ± 0.028	4.26 ± 0.017
	Tibia length	Components of PC axis “leg size” abbreviated LS	9.01 ± 0.050	9.49 ± 0.086	9.34 ± 0.064	9.48 ± 0.051	9.28 ± 0.034
	Femur length		8.20 ± 0.042	8.74 ± 0.065	8.54 ± 0.044	8.53 ± 0.042	8.44 ± 0.028
	Femur width		1.47 ± 0.007	1.50 ± 0.016	1.47 ± 0.016	1.47 ± 0.009	1.47 ± 0.005

### Statistical analysis

All statistics were carried out using R version 3.0.2 (R Development Core Team 2013). First, to see whether sampling years should be analyzed separately, I tested whether there were any significant interaction effects of year on the relationship between traits and fitness. I started with a saturated model that included all traits, quadratic trait terms, sampling date, and all interactions between traits and year. I then simplified the model using backwards stepwise model selection, and averaged the coefficients of terms where models had ΔAIC < 5.

To investigate the relationship between measured traits and fitness in male and female tree crickets in each year, I used several methods: Firstly, I conducted a cubic spline analysis (Schluter 1988; Schluter and Nychka 1994) to visualize selection. To do this, I conducted a principal component analysis to reduce the measured traits to a single PC axis. I then fit a cubic spline (in a generalized additive model) to the relationship between the PC trait (with the same original trait composition for all years and both sexes) and my estimate of fitness, and plotted this relationship for each sex in each year. Secondly, I calculated standardized selection differentials, which show, in standard deviations, how trait size has changed after selection (Arnold and Wade 1984). This was calculated as the covariance between fitness and standardized trait sizes. These values include phenotypic change as a result of both direct and indirect selection on that trait. I also calculated selection gradients (Lande and Arnold 1983) from multiple regression of standardized traits against my estimate of fitness. This term measures only the force of direct selection on that trait (Arnold and Wade 1984). Fitness was estimated as a score 0 if the cricket was prey of the predatory wasp and 1 if the cricket was a survivor sampled from the remaining population. I did not convert absolute fitness to relative fitness because converting to relative fitness gives the false impression that I sampled survivors and prey in proportion to the frequency in which they were hunted by wasps. For each year and sex, I found linear selection gradients (*β*) using multiple linear regression (which included only linear terms), and quadratic and correlational selection gradients (**γ**) from separate multiple regression models that included quadratic and cross‐product terms. Quadratic selection gradients were obtained by doubling the quadratic coefficients from nonlinear regression (Stinchcombe et al. 2008). As the estimate of fitness was binary, I used logistic regression to generate *P*‐values of regression coefficients (Janzen and Stern 1998). Finally, I conducted canonical analyses to increase the ability to detect nonlinear and correlational selection (Phillips and Arnold 1989; Blows and Brooks 2003). This consisted of multiplying the matrix of standardized trait measurements by the matrix **M** (the diagonalization of the **γ**‐matrix) to obtain composite traits, and conducting a second round of linear and nonlinear regression on these composite traits. Significance of eigenvalues generated by canonical analysis were found using multiple permutation tests (Reynolds et al. 2009), and cross‐product terms were added back into the model for the permutation test (Bisgaard and Ankenman 1996). If convex or concave selection was detected, I tested whether that selection was significantly stabilizing or disruptive (respectively) (Mitchell‐Olds and Shaw 1987) using MOStest function in the R package “vegan”(Oksanen et al. 2012). To test whether directional, quadratic, and correlational selection was significantly different between males and females each year, I conducted partial *F*‐tests (Chenoweth and Blows 2005). This consisted of conducting an analysis of variance on models of selection on all traits with and without sex as an interaction term. I also used similar partial *F*‐tests to compare the difference in selection between pairs of years of this study. Significance values of partial *F*‐tests were obtained by permutation tests.

## Results

### Selection over 4 years

**Figure 1:**
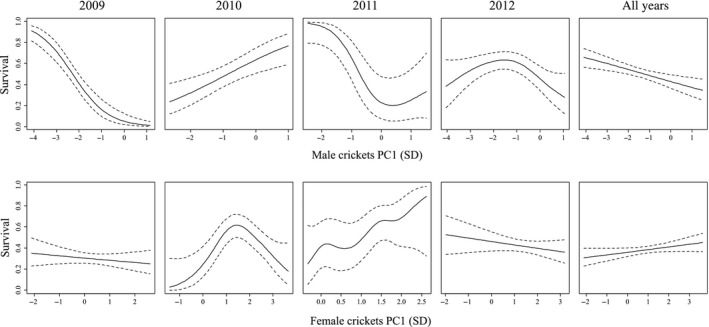
Cubic spline visualizations of viability selection on male and female *Oecanthus nigricornis* phenotypic traits between 2009 and 2012. The source of viability selection was predation by solitary wasp *Isodontia mexicana*. Traits were compressed into one principal component axis which explains 69% of trait variance. Relative trait loadings are the same for males and females. The solid line represents the fitted polynomial, and the dotted lines indicate 95% confidence intervals.

Cubic spline analysis showed that the form of viability selection on principal component axis 1 was considerably different from year to year among both males and females (Fig. 1). PC axis 1 captured 69% of total variance and traits influenced this axis in the following proportion: tegmen width −0.35; pronotum length 0.53; head width 0.55; leg size 0.54. Selection on PC1 in males was especially variable, as it changed from negative linear to positive linear to concave to convex from 2009 to 2012. Partial *F*‐tests support that selection on males varied from year to year: Linear selection on male traits was significantly different between 2009 and 2010 as well as between 2010 and 2011 (Table 2a). Cubic spline analysis suggests that the shape of selection on females changes considerably from year to year (Fig. 1), but these differences are not statistically significant (partial *F*‐tests: Table 2a).

**Table 2:** Results of analysis of deviance tests comparing (a) selection between the 4 years of this study, and (b) results of selection analyses between male and female *Oecanthus nigricornis*

(a)		
	Is selection different from previous year?	
	Males	Females
2009–2010		
Linear	**Yes** (*F *=* *3.29, *P* < 0.01)	No (*F* = 0.11, *P* = 0.99)
Quadratic	No (*F *=* *0.20, *P* = 0.94)	No (*F* = 1.25, *P* = 0.34)
Correlational	No (*F *=* *0.66, *P* = 0.64)	No (*F* = 1.54, *P* = 0.23)
2010–2011		
Linear	**Yes** (*F *=* *2.59, *P* = 0.03)	No (*F* = 1.36, *P* = 0.26)
Quadratic	No (*F *=* *0.11, *P* = 0.98)	No (*F* = 1.00, *P* = 0.43)
Correlational	No (*F *=* *1.04, *P* = 0.40)	No (*F* = 0.96, *P* = 0.46)
2011–2012		
Linear	No (*F* = 1.57, *P* = 0.12)	No (*F* = 1.74, *P* = 0.10)
Quadratic	No (*F* = 1.12, *P* = 0.36)	No (*F* = 0.25, *P* = 0.93)
Correlational	No (*F* = 1.05, *P* = 0.40)	No (*F* = 0.73, *P* = 0.63)

(b)		
Type of selection	Is it different between males and females?	
2009		
Linear	**Yes** (*F *=* *2.86, *P *=* *0.01)	
Quadratic	No (*F *=* *0.78, *P *=* *0.57)	
Correlational	No (*F *=* *0.29, *P *=* *0.90)	
2010		
Linear	No (*F *=* *0.67, *P *=* *0.69)	
Quadratic	No (*F *=* *0.43, *P* = 0.79)	
Correlational	No (*F *=* *1.06, *P *=* *0.39)	
2011		
Linear	No (*F *=* *2.38, *P *=* *0.21)	
Quadratic	No (*F *=* *0.19, *P *=* *0.94)	
Correlational	No (*F *=* *0.32, *P *=* *0.86)	
2012		
Linear	**Yes** (*F *=* *2.42, *P *=* *0.03)	
Quadratic	No (*F *=* *2.16, *P *=* *0.08)	
Correlational	No (*F *=* *0.64, *P *=* *0.64)	
All 4 years		
Linear	No (*F *=* *1.80, *P *=* *0.10)	
Quadratic	No (*F *=* *1.11, *P *=* *0.36)	
Correlational	No (*F *=* *0.94, *P *=* *0.45)	

**Figure 2:**
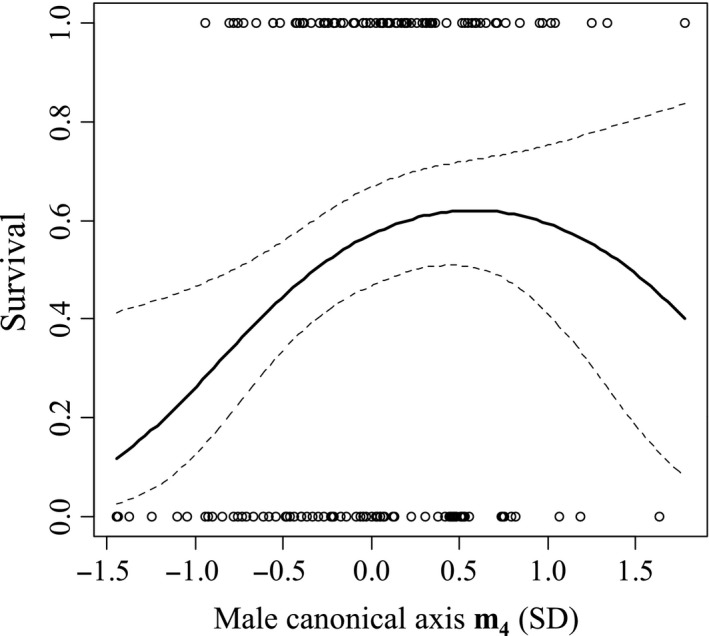
Cubic spline visualization of significant viability selection on male composite trait **m**_**4**_ over a 4‐year period. The solid line represents the fitted polynomial, and the dotted lines indicate 95% confidence intervals.

**Figure 3:**
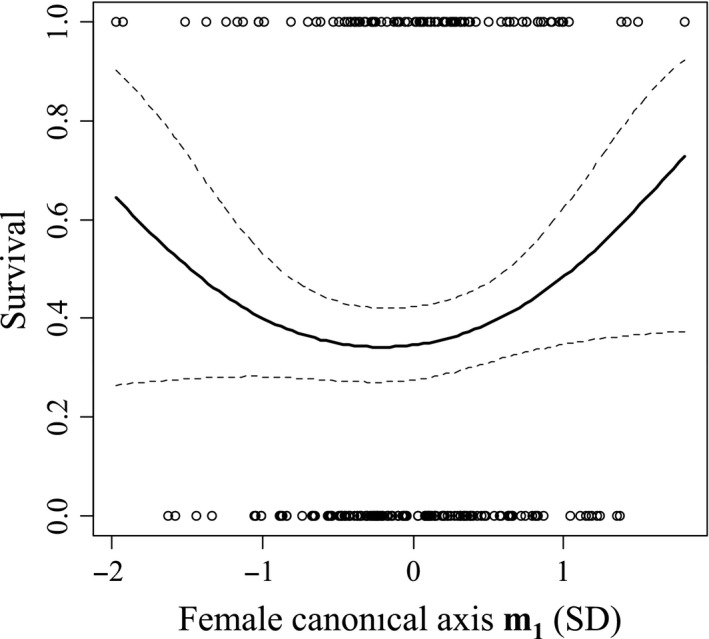
Cubic spline visualization of significant viability selection on female composite trait **m**_**1**_ over a 4‐year period. The solid line represents the fitted polynomial, and the dotted lines indicate 95% confidence intervals.

Viability selection analyzed across the entire 4‐year period was weaker than in individual years for both males and females. The mean magnitude of directional selection gradients within years (calculated by averaging the absolute values of yearly *β* on individual traits in Table 3) was more than double the magnitude of that across the 4 years (the average of absolute values of *β* on individual traits over the entire 2009–2012 period) in both males (${\overset{¯}{x}}_{\beta -\mathrm{within}-\mathrm{year}}=0.190$, ${\overset{¯}{x}}_{\beta -\mathrm{across}-\mathrm{year}}=0.092$, two‐tailed *t*‐test, *t*_18_ = 3.16, *P *<* *0.01) and females (${\overset{¯}{x}}_{\beta -\mathrm{within}-\mathrm{year}}=0.098$, ${\overset{¯}{x}}_{\beta -\mathrm{across}-\mathrm{year}}=0.025$, two‐tailed *t*‐test, *t*_18_ = 2.61, *P *=* *0.02). Over the 4‐year period, there was no significant linear selection on original traits in either sex (Table 3), but canonical analysis (Table 4) revealed selection on composite traits. Canonical analysis found significant linear (*θ *= 0.162, *P *=* *0.02) and nonlinear selection (*λ *= −0.385, *P *=* *0.03) on male composite trait **m**_**4**_ (influenced by leg size and tegmen width) resulting in males with moderately larger tegmina and smaller legs having a survival advantage (Fig. 2). Canonical analysis also found concave selection on a female composite trait **m**_**1**_ (strongly influenced by head width, *λ* *= *0.230, *P *=* *0.03), which resulted in a fitness trough for females with intermediate head widths (Fig. 3). Cubic spline analysis of selection of males and females over the same period shows weak linear selection in opposite directions (Fig. 1). This indicates that there is nonsignificant (*P *=* *0.09), negative selection on male pronotum length, head width, and leg size, and positive selection on wing width (as wing width is loaded in opposition to the other traits on this axis). There is nonsignificant (*P *=* *0.37), weak positive selection on the same trait axis in females. It is important to note that the relative trait loadings of the axis PC1 are different than those of both male axis **m**_**4**_ and female axis **m**_**1**_, which is why they show different relationships between fitness and phenotype. The axis PC1 combines trait values across the 4 years and both sexes in a manner that captures the most variance, whereas axes from canonical rotation were calculated to show the combinations of traits under the strongest nonlinear selection within each period of selection for males and females, respectively. Thus the relative trait loadings of canonical axes under significant selection changed considerably from year to year.

**Table 3:** Vectors of standardized directional selection gradients (*β*) (and their associated standard errors) and selection differentials (S) for viability selection on male and female *Oecanthus nigricornis* over 4 years

Males				Females			
	*S*	*β*	SE		*S*	*β*	SE
2009 (23 Survivor, 28 Prey)				2009 (28 Survivor, 66 Prey)			
PL	−**0.202**	−0.134	0.087	PL	−0.039	−0.073	0.08
HW	−0.124	0.090	0.090	HW	0.017	0.059	0.06
LS	−**0.264**	−**0.298**	0.079	LS	−0.023	−0.029	0.08
TW	0.050	**0.165**	0.064	TW	0.029	0.055	0.06
2010 (14 Survivor, 14 Prey)				2010 (18 Survivor, 23 Prey)			
PL	0.18	0.134	0.164	PL	0.011	−0.042	0.13
HW	0.17	0.158	0.138	HW	0.054	0.073	0.12
LS	0.12	−0.207	0.178	LS	0.028	0.003	0.14
TW	**0.20**	0.157	0.138	TW	0.012	0.019	0.10
2011 (9 Survivor, 5 Prey)				2011 (10 Survivor, 8 Prey)			
PL	−0.048	0.118	0.17	PL	0.255	0.430	0.20
HW	−0.385	−**0.556**	0.16	HW	0.024	−0.222	0.15
LS	−0.157	0.151	0.24	LS	0.177	0.048	0.21
TW	0.142	−0.013	0.12	TW	0.074	−0.173	0.15
2012 (30 Survivor, 24 Prey)				2012 (34 Survivor, 46 Prey)			
PL	−0.025	0.209	0.14	PL	−0.044	−0.114	0.10
HW	−**0.142**	−**0.261**	0.11	HW	−0.047	−0.063	0.09
LS	−0.003	0.158	0.12	LS	0.011	0.120	0.10
TW	−**0.153**	−**0.226**	0.10	TW	0.033	0.052	0.06
All years (76 Survivor, 71 Prey)				All years (90 Survivor, 143 Prey)			
PL	−0.024	0.086	0.07	PL	0.027	−0.019	0.06
HW	−**0.086**	−0.107	0.06	HW	0.019	−0.005	0.04
LS	−0.073	−0.105	0.07	LS	0.037	0.027	0.06
TW	0.000	0.070	0.05	TW	0.054	0.051	0.04

Bolded values are significant at *α *= 0.05. None of the selection differential or gradients were significant, thus none are bolded.

**Table 4:** Comparison of linear (*θ*) and nonlinear (*λ*) viability selection on canonical trait axes in male and female *Oecanthus nigricornis* over 4 years (lacking results from 2011, as too few adults were collected to perform a canonical analysis)

	Males							Females					
	PL	HW	LS	TW	*θ*	*λ*		PL	HW	LS	TW	*θ*	*λ*
2009													
**m**_**1**_	0.913	−0.154	−0.250	−0.282	−0.108	0.298	**m**_**1**_	−0.497	0.781	−0.111	0.361	0.105	0.217
**m**_**2**_	0.406	0.294	0.518	0.693	−0.068	−0.011	**m**_**2**_	0.516	0.538	0.612	−0.266	−0.039	0.015
**m**_**3**_	0.036	0.451	0.599	−0.661	−**0.252**	−0.072	**m**_**3**_	0.203	−0.216	0.398	0.868	0.009	−0.060
**m**_**4**_	0.007	0.007	−0.556	0.061	**0.249**	−0.468	**m**_**4**_	0.668	0.232	−0.675	0.211	−0.004	−0.542
2010													
**m**_**1**_	0.150	−0.639	0.753	−0.043	−0.244	0.823	**m**_**1**_	0.840	−0.105	−0.261	−0.464	−0.053	**1.615**
**m**_**2**_	0.143	0.630	0.537	0.543	0.092	0.088	**m**_**2**_	0.389	−0.312	0.804	0.323	−0.031	0.123
**m**_**3**_	0.861	−0.204	−0.326	0.332	0.203	−0.343	**m**_**3**_	0.153	−0.475	−0.533	0.684	−0.030	−0.261
**m**_**4**_	−0.464	−0.391	−0.196	0.770	0.037	−0.821	**m**_**4**_	0.345	0.816	−0.037	0.462	0.053	−**0.341**
2012													
**m**_**1**_	0.102	−0.726	0.648	−0.207	**0.361**	0.541	**m**_**1**_	0.729	−0.357	−0.583	−0.039	−0.133	**0.810**
**m**_**2**_	−0.147	0.106	0.426	0.886	−0.191	0.316	**m**_**2**_	−0.194	0.221	−0.435	0.851	0.000	0.232
**m**_**3**_	0.423	0.647	0.569	−0.281	0.073	−**0.291**	**m**_**3**_	0.653	0.546	0.465	0.245	−0.041	−0.001
**m**_**4**_	0.888	−0.207	−0.275	0.304	0.128	−**0.518**	**m**_**4**_	−0.074	0.725	−0.504	−0.463	−0.121	−0.211
All years													
**m**_**1**_	0.819	−0.381	0.031	−0.428	0.078	0.313	**m**_**1**_	−0.478	0.744	−0.248	0.395	0.019	**0.230**
**m**_**2**_	0.010	0.535	−0.677	−0.506	−0.021	0.273	**m**_**2**_	−0.093	0.467	0.665	−0.575	−0.012	0.048
**m**_**3**_	0.503	0.725	0.326	0.340	−0.045	−0.034	**m**_**3**_	0.552	0.183	0.503	0.640	0.035	−0.018
**m**_**4**_	0.277	−0.208	−0.659	0.667	**0.162**	−**0.385**	**m**_**4**_	0.677	0.440	−0.494	−0.322	−0.045	−0.234

The **M**‐matrix of relative loadings of the original traits on the new canonical axes is also included. Bolded values indicate significance at *α *= 0.05).

### Differences in selection on males and females

Cubic spline analysis indicated that the shape of phenotypic selection each year was quite different between males and females (Fig. 1). Selection on PC1 was strongly negative in males in 2009, but almost flat in females (Fig. 1), and partial *F*‐tests confirm that linear selection was significantly different between males and females in 2009 (*F = *2.86, *P *=* *0.01, Table 2b). Partial *F*‐tests also indicate that directional selection was significantly different (*F *=* *2.42, *P *=* *0.03) and quadratic selection was marginally different (*F *=* *2.16, *P *=* *0.08) in 2012. We can see from the cubic splines that selection on PC1 in 2012 was weakly negative in females, but was strongly convex and almost stabilizing in males.

In general, nonlinear selection was much more common than linear in females, and linear selection was more common than nonlinear in males (Tables 3 and 4).

### Yearly viability selection on males

There were significant interactions between year and the male traits of head width, leg size, wing width, pronotum size (Supplementary Table S1a), so each year was analyzed separately.

Among males, linear selection was frequently detected, but the traits under significant selection changed every year. In 2009 males, significant directional selection for smaller legs (*β = *−0.298, *P* = 0.01) and wider tegmina (*β = *−0.298, *P *=* *0.01) was detected. Significant selection differentials indicate a reduction in the pronotum length (*S *=* *−0.202, *P *=* *0.01) and leg size (*S *=* *−0.264, *P *<* *0.01) in 2009. After canonical analysis, I found significant linear selection on composite traits **m**_**3**_ (for larger tegmen and smaller legs, *θ *= −0.252, *P *<* *0.01) and **m**_**4**_ (for wider heads and smaller legs, *θ *= 0.249, *P *=* *0.02, Table 4). In 2010, there was no significant selection gradients before or after canonical rotation, but a significant selection differential indicated that tegmen width increased after predation (*S *=* *0.203, *P *=* *0.05, Table 3). In 2011, there was significant directional selection for narrower heads (*β *= −0.556, *P *=* *0.01). I could not conduct any nonlinear analyses in 2011 because only five adult male *O. nigricornis* were found in wasp nests. In 2012, significant selection differentials and gradients detected selection for narrower heads (*S *=* *−0.142, *P *=* *0.04, *β = *−0.261, *P *=* *0.02) and tegmina (*S *=* *−0.153, *P *=* *0.03, *β = *−0.226, *P *=* *0.03, Table 3). After canonical analysis, I detected linear selection on composite traits **m**_**1**_ (for narrower heads and larger legs, *θ *= 0.361, *P *=* *0.01). I also found nonlinear convex selection on **m**_**3**_ (mostly influenced by head width and leg size, *λ *= −0.291, *P *<* *0.01) and **m**_**4**_ (pronotum length, *λ *= −0.518, *P *=* *0.02, Table 4).

Directional viability selection on males did not consistently predict changes in trait size in the next generation (Tables 1 and 2). In 2011, significant selection for narrower heads was detected, and 2012 males had significantly narrower heads ($\overset{¯}{x}$_2011_ = 1.61 mm $\overset{¯}{x}$_2012_ = 1.54 mm, two‐tailed *t*‐test, *t*_66_ = 2.49, *P *=* *0.02). However, in 2009, I saw significant selection for smaller pronotum length and leg size, yet in 2010, male pronota and legs were significantly larger ($\overset{¯}{x}$_2009_ = 2.20 mm $\overset{¯}{x}$_2010_ = 2.37 mm, two‐tailed *t*‐test, *t*_77_ = −5.39, *P *<* *0.01).

### Yearly viability selection on females

As with males, I found significant interactions between a trait (tegmen width) and sampling year in selection on females, so I analyzed selection separately for each year (Supplementary Table S1b). There were no significant selection differentials or directional selection gradients on any original female traits in any year (Table 3), but canonical analysis did reveal nonlinear selection on composite female traits (Table 4). Although the relative trait loadings for canonical traits changed considerably from year to year (see **M**‐matrices, Table 4), composite traits that mostly represented pronotum length were frequently under significant selection. In 2009, there was marginally significant nonlinear convex selection on composite trait **m**_**4**_ (*λ *= −0.542, *P *=* *0.06), which represented pronotum length and leg size equally. In 2010, there was significant concave (*λ = *1.615, *P *=* *0.01) selection on composite trait **m**_**1**_ (which is strongly influenced by pronotum length) and significantly convex (*λ *= −0.341, *P *<* *0.01) and stabilizing selection (Mitchell‐Olds Shaw test, *P *=* *0.03) on **m**_**4**_ (mostly representing head width). In 2011, I could not conduct a canonical analysis on female traits because only eight adult females were found in *I. mexicana* nests. In 2012, I found significantly concave (*λ *= 0.810, *P *=* *0.01) and disruptive (*P *=* *0.02) selection on composite trait **m**_**1**_, which mostly represents pronotum length.

## Discussion

The magnitude of viability selection gradients on both male and female *O. nigricornis* within years was larger than across years (Table 3). Over the 4‐year period, there were no significant directional selection gradients on any original traits in both sexes. Males were subject to significant directional selection within years, but which traits were under selection changed significantly from year to year, resulting in no significant selection gradients on any one trait over the 4‐year period. However, a significant selection differential shows that male head width became slightly narrower over the 4 years. Similar temporal variation in selection is commonly observed in long‐term selection studies (e.g., Kalisz 1986; Gibbs and Grant 1987; Milner et al. 1999; Punzalan et al. 2010; Siepielski et al. 2011), and this variation may dampen the strength of directional selection (Chaine and Lyon 2008; Siepielski et al. 2009); but also see Morrissey and Hadfield 2012).

Yearly variation in selection instead resulted in significant nonlinear selection on both males and females over the 4‐year period (Table 4). Males experienced significant convex selection on composite trait **m**_**4**_ that resulted in males with relatively larger tegmina and smaller legs having a survival advantage against predatory wasps, but this advantage diminishes as the trait value increases (Fig. 2). These results contrast with the findings in Ercit and Gwynne (2015) that males with smaller tegmina and larger legs had a survival advantage in 2012. However, as the form of total selection over 4 years on the tegmen/legs size trait is convex, male crickets from 2012 may have had larger‐than‐average traits on axis **m**_**4**_ and may represent the downward slope seen on the right side of the graph in Figure 2. Females experienced concave selection on composite trait **m**_**1**_ that indicates a fitness trough for females with intermediate head widths, and females with large and small head widths are more likely to survive wasp predation. This selection appears to be disruptive (Fig. 3), but it is not significantly so. It is not clear why female head width is important in viability selection over the 4 years, especially as I did not detect significant selection on it within any single year. Selection on female head width may be a statistical artifact, or it may be that head width in females is more strongly correlated to body mass than estimated, and concave selection on head width may result from disruptive selection on body mass. Cubic spline analysis and partial *F*‐tests showed that the shape and direction of viability selection were different between males and females within years. However, total selection over the 4‐year period was not significantly different between males and females. Such temporal variation in the difference in selection between males and females may slow the evolution of sexual dimorphism.

It is interesting that males experienced mostly linear selection, whereas for females it was mainly nonlinear. Linear viability selection on males may be connected to sexual selection on males: The results of Ercit and Gwynne (2015) show that traits that made males successful at mating also made them more likely to be killed by wasps in 2012. If mating per se is risky for males, male traits that attract females will also be subject to viability selection by *I. mexicana*. If this sexual selection is mostly linear (as it predominantly was in 2012 [Ercit and Gwynne 2015; ]), and if predation risk in males increases linearly with mating success, we may expect opposing viability selection on males to also be linear. If sexually attractive males attract more predators, this may also explain why male traits under viability selection change from year to year. In other animals, the male traits that are related to mating success can vary between years (e.g., Hughes et al. 1999; Chaine and Lyon 2008). If this is the case in tree crickets, the traits of successfully mating males would vary between years, and so might the traits of males killed in risky mating behaviors.

The observed nonlinear selection on females may be due to biases and limitations of the predator. In 2010 and 2012 females, I saw significant concave selection on composite traits that represent body size (pronotum length), which can be expected based on results from (Ercit 2014): *I. mexicana* take large prey, but the largest females may be too heavy for the wasp to transport (Marden 1987; Coelho and Ladage 1999). Thus, the observed temporal variation in viability selection may be caused partly by variation in predator size, possibly exacerbated by sampling error, as I only sampled prey from the few dozen wasps that nested in the trap nests each year.

One limitation of this study was that I was not able to estimate what proportion of the population was killed by wasp predation each year. Variation in relative predator and prey populations could significantly affect the intensity of viability selection (Benkman 2013). In the presence of abundant nesting habitat, some solitary sphecids can have significant impacts on prey density (Dukas 2005). If wasps overhunt one prey population, some solitary wasps will switch prey species (Polidori et al. 2007), and indeed, I observed such prey‐switching during the course of this study. In 2011, part of the reason why so few *O. nigricornis* were collected was because most wasps were provisioning other *Oecanthus* species. Thus, the intensity of predation by wasps on this study population of *O. nigricornis* likely varied greatly from year to year, and in turn, affected the intensity of selection.

Although males in our population experienced significant directional selection on traits, I did not reliably see significant change in that trait in the next generation. This result is not surprising, as I have only measured selection from one component of fitness – viability selection from a single predator. Tree crickets also experience viability selection from other predators such as spiders and birds, as well as from environmental factors. Furthermore, within a generation, viability selection may be counteracted by fecundity or sexual selection (as in Ercit and Gwynne 2015). Even if total natural selection (selection from every component of fitness) was significantly directional, this selection may be acting on phenotypic variance caused by environmental rather than heritable variables, as was found in a study of collared flycatchers (Alatalo et al. 1990). In another multigenerational study, Milner et al. (1999) found repeated selection for larger body weight in Soay sheep did not result in any change in population mean weight, and this was likely due to selection acting on phenotypic variance caused by the environment.

The results presented here are consistent with other multigenerational studies of selection that show that the direction of viability selection is variable between generations (summarized in Siepielski et al. 2011). The results of this study underline the importance of temporal scale in selection studies. If short‐term studies are extrapolated to long‐term selection (Hoekstra et al. 2001), this may overestimate the rates of evolution and sexual dimorphism (Kinnison and Hendry 2001). Thus, this study adds to our knowledge of how selection acts in different timescales on a natural population and may help in future studies to estimate rates of evolution in nature.

## Conflict of Interest

None declared.

## Supporting information


**Appendix S1.** Supplementary tables.
**Table S1.** Results of AIC model selection and multi‐model averaging of viability selection models in a) male and b) female *Oecanthus nigricornis*.
**Table S2.** Gamma matrices of quadratic and correlational selection gradients for viability selection on male and female *Oecanthus nigricornis* between 2009 and 2012. Bolded terms indicate significance at *α* = 0.05.

**Data S1.** Morphological measurements and viability status for all crickets sampled for this study.
