# Supplementary material for: Temporal variation in selection on male and female traits in wild tree crickets
Source: Ecol Evol. 2016 Jun 16;6(15):5118–28. doi: 10.1002/ece3.2105 (PMC4984491; doi:10.1002/ece3.2105)
Supplement: Supplementary file 1 — Appendix S1. Supplementary tables. Table S1. Results of AIC model selection and multi‐model averaging of viability selection models in a) male and b) female Oecanthus nigricornis. Table S2. Gamma matrices of quadratic and correlational selection gradients for viability selection on male and female Oecanthus nigricornis between 2009 and 2012. Bolded terms indicate significance at α = 0.05. [file ECE3-6-5118-s001.docx]

Appendix S1: Supplementary Tables

**Supplementary Table S1**

Results of AIC model selection and multi-model averaging of viability selection models in a) male and b) female *Oecanthus nigricornis*.

a) *Males*

| Term | Full model-averaged coefficient | P |
| --- | --- | --- |
| Year | 0.054 | 0.22 |
| PL | -235.4 | 0.07 |
| **HW** | **267.3** | **0.01** |
| **LS** | **-365.0** | **0.00** |
| **TW** | **326.8** | **0.00** |
| **Sampling date** | **0.009** | **0.01** |
| **PL^2^** | **181.8** | **0.02** |
| HW^2^ | -19.72 | 0.67 |
| TW^2^ | -118.7 | 0.06 |
| PL*TW | 184.8 | 0.12 |
| PL*Year | 0.117 | 0.07 |
| **HW*Year** | **-0.133** | **0.01** |
| **LS*Year** | **0.182** | **0.00** |
| **TW*Year** | **-0.163** | **0.00** |
| **PL^2^*Year** | **-0.090** | **0.02** |
| TW^2^*Year | 0.059 | 0.06 |
| PL*TW*Year | -0.092 | 0.12 |
| HW^2^*Year | 0.010 | 0.67 |
| LS^2^ | -0.002 | 0.87 |
| HW*LS | 0.001 | 0.94 |

b) *Females*

| Term | Full model-averaged coefficient | p |
| --- | --- | --- |
| Year | -0.011 | 0.75 |
| Sampling date | 0.006 | 0.10 |
| **TW^2^** | **-71.07** | **0.05** |
| **TW^2^*Year** | **0.035** | **0.05** |
| LS | 0.007 | 0.81 |
| PL | -0.005 | 0.84 |
| PL*LS | -0.002 | 0.86 |

**Supplementary Table S2**

Gamma matrices of quadratic and correlational selection gradients for viability selection on male and female *O. nigricornis* between 2009 and 2012. Bolded terms indicate significance at α = 0.05.

|  | **Males** |  |  |  |  | **Females** |  |  |  |
| --- | --- | --- | --- | --- | --- | --- | --- | --- | --- |
| **2009** | **γ-matrix** |  |  |  |  | **γ-matrix** |  |  |  |
|  | **PL** | **HW** | **LS** | **TW** |  | **PL** | **HW** | **LS** | **TW** |
| **PL** | 0.246 | - | - | - |  | -0.186 | - | - | - |
| **HW** | -0.047 | -0.330 | - | - |  | -0.161 | 0.105 | - | - |
| **LS** | -0.070 | 0.206 | -0.155 | - |  | 0.256 | 0.076 | -0.248 | - |
| **TW** | -0.078 | 0.008 | 0.062 | -0.015 |  | -0.128 | 0.044 | 0.045 | -0.040 |
| **2010** | **γ-matrix** |  |  |  |  | **γ-matrix** |  |  |  |
|  | **PL** | **HW** | **LS** | **TW** |  | **PL** | **HW** | **LS** | **TW** |
| **PL** | -0.411 | - | - | - |  | 1.111 | - | - | - |
| **HW** | -0.160 | 0.231 | - | - |  | -0.234 | -0.256 | - | - |
| **LS** | 0.122 | -0.452 | 0.424 | - |  | -0.290 | -0.043 | 0.115 | - |
| **TW** | 0.197 | 0.323 | 0.160 | -0.497 |  | **-0.695** | 0.023 | 0.328 | 0.166 |
| **2012** | **γ-matrix** |  |  |  |  | **γ-matrix** |  |  |  |
|  | **PL** | **HW** | **LS** | **TW** |  | **PL** | **HW** | **LS** | **TW** |
| **PL** | -0.448 | - | - | - |  | 0.437 | - | - | - |
| **HW** | -0.030 | 0.145 | - | - |  | -0.210 | 0.003 | - | - |
| **LS** | 0.072 | -0.377 | 0.151 | - |  | -0.333 | 0.223 | 0.266 | - |
| **TW** | -0.158 | 0.197 | 0.137 | 0.201 |  | -0.069 | 0.126 | -0.117 | 0.124 |
| **All years** | **γ-matrix** |  |  |  |  | **γ-matrix** |  |  |  |
|  | **PL** | **HW** | **LS** | **TW** |  | **PL** | **HW** | **LS** | **TW** |
| **PL** | 0.172 | - | - | - |  | -0.060 | - | - | - |
| **HW** | -0.086 | 0.089 | - | - |  | **-0.155** | 0.092 | - | - |
| **LS** | 0.071 | -0.163 | -0.046 | - |  | 0.098 | 0.022 | -0.026 | - |
| **TW** | **-0.188** | 0.022 | **0.255** | -0.048 |  | 0.004 | 0.086 | -0.084 | 0.020 |
